# Human Wharton’s Jelly Stem Cell Secretions Inhibit Human Leukemic Cell Line K562 *in vitro* by Inducing Cell Cycle Arrest and Apoptosis

**DOI:** 10.3389/fcell.2021.614988

**Published:** 2021-03-18

**Authors:** Muneerah A. H. Huwaikem, Gauthaman Kalamegam, Ghadeer Alrefaei, Farid Ahmed, Roaa Kadam, Talal Qadah, Khalid H. W. Sait, Peter N. Pushparaj

**Affiliations:** ^1^Department of Medical Laboratory Technology, Faculty of Applied Medical Sciences, King Abdulaziz University, Jeddah, Saudi Arabia; ^2^Department of Medical Laboratory Technology, Faculty of Applied Medical Sciences, University of Tabuk, Tabuk, Saudi Arabia; ^3^Stem Cells Unit, Centre of Excellence in Genomic Medicine Research (CEGMR), King Abdulaziz University, Jeddah, Saudi Arabia; ^4^Biology Department, Faculty of Sciences, University of Jeddah, Jeddah, Saudi Arabia; ^5^Embryonic and Cancer Stem Cell Research Group, King Fahad Medical Research Center, King Abdulaziz University, Jeddah, Saudi Arabia; ^6^Department of Obstetrics and Gynaecology, King Abdulaziz University, Jeddah, Saudi Arabia

**Keywords:** chronic myeloid leukemia, human Wharton’s jelly stem cells, cell cycle, apoptosis, gene expression, cytokines

## Abstract

Emerging resistance to the tyrosine kinase inhibitors that target the BCR-ABL1 oncoprotein has prompted research for novel therapeutics against chronic myeloid leukemia (CML). Herein, we evaluated the tumor inhibitory properties of the human Wharton’s jelly stem cells (hWJSCs) co-culture (hWJSC-CC) and their extracts, namely, the hWJSC-conditioned medium (hWJSC-CM; 100%) and hWJSC-lysate (hWJSC-L; 15 μg/ml), on a CML cell line K562 *in vitro*. The hWJSCs expressed mesenchymal stem cell (MSC)-related cluster of differentiation (CD) markers and demonstrated mesodermal tissue differentiation potential. The cell metabolic activity showed a mean maximal decrease in the K562 cells by 49.12, 41.98, and 68.80% following treatment with the hWJSC-CC, hWJSC-CM, and hWJSC-L, respectively, at 72 h. The sub-G1 population in the cell cycle was decreased by 3.2, 4.5, and 3.8% following treatment with the hWJSC-CC, hWJSC-CM, and hWJSC-L, whereas the G2/M cell population was increased by 13.7 and 12.5% with the hWJSC-CM and hWJSC-L, respectively, at 48 h. Annexin V–allophycocyanin (APC) assay showed an increase in the apoptotic cells by 4.0, 3.9, and 4.5% at 48 h. The expression of pro-apoptotic *BAX* and *CASP3* genes were increased, whereas BIRC5 (*Survivin*) was decreased compared with the control. The pro-inflammation-related genes, namely, *IFN-γ*, *TNF-α*, *IL-1β*, *IL-6*, *IL-8*, and *IL-12A*, were decreased, whereas the anti-inflammatory genes, namely, *IL-4* and *IL-10*, were increased following treatment with the hWJSC-CC, hWJSC-CM, and hWJSC-L at 48 h. Multiplex bead-based cytokine assay also demonstrated decreases in the pro-inflammatory cytokines (IFN-γ, TNF-α, IL-1β, IL-6, and IL-12) and an increase in the anti-inflammatory cytokine (IL-10) compared with the control. The pro-inflammatory cytokine IL-8 showed an increase with the hWJSC-CC and decreases with both the hWJSC-CM and the hWJSC-L. The hWJSCs and their extracts inhibited the K562 cells by causing cell cycle arrest and inducing apoptosis *via* the soluble cellular factors. However, an *in vivo* evaluation is necessary to unravel the true potential of the hWJSCs and their extracts before its use in CML inhibition.

## Introduction

Leukemia is a heterogeneous group of hematopoietic cancers that belong to either myeloid or lymphoid lineages. The global burden of disease cancer collaboration study identified leukemia as having the eighth highest incidence, with 606,000 new cases and 35,300 deaths (Wang et al., 2016). The majority of the leukemic disorders arise as a *de novo* malignancy in an otherwise healthy individual and are marked by clonal expansion of undifferentiated myeloid precursors, with the production of abnormal cells. Among the various adult leukemias, chronic myeloid leukemia (CML) is a chronic myeloproliferative disorder and accounts for 15% of the adult leukemia (American Cancer Society, 2015). The Saudi cancer registry reported 8,712 cases of leukemia during the study period 1999–2013, with a higher incidence in males (57.2%) than in females (42.8%). In contrast to the global higher incidence in adults, in Saudi Arabia, the highest age-standardized rate was reported in children aged 0–4 years (Bawazir et al., 2019).

CML is characterized by chromosomal translocation of “breakpoint cluster region, *bcr*” and “Abelson, *abl*” gene sequences from chromosomes 22 and 9, respectively, *t*(9;22)(q34;q11.2), well known as Philadelphia (Ph) translocation or Ph chromosome (Nowell and Hungerford, 1960). Rearrangement and amplification by 4–8-fold of cellular *abl* sequences were reported in K562, a Ph-positive cell line derived from a patient with CML in blast crisis (Collins and Groudine, 1983). The Bcr-Abl active tyrosine kinase is responsible for the maintenance of the malignant phenotype and also the intracellular signaling related to cell survival and proliferation (Kabarowski and Witte, 2000). Abnormal cellular expansion or malignant hematopoiesis occurs at the expense of normal hematopoietic mechanisms and hence is associated with a decrease in mature cells, immunosuppression, and marrow failure (Bonnet and Dick, 1997).

Over the last two decades, the management of CML mainly relies upon the tyrosine kinase inhibitors (TKIs), such as the first generation (imatinib), the second generation (nilotinib, dasatinib, bosutinib), and/or its third generation (ponatinib). The effectiveness of these therapies and the overall survival rates in CML are based upon the complete hematologic, cytologic, or molecular responses to these pharmacological agents (Bonifacio et al., 2019). The emergence of resistance with frontline TKIs has made the targeted therapies to become less effective especially when disease transition occurs from chronic to accelerated or blast crisis (Goldman and Melo, 2003; Bonifacio et al., 2019). Nevertheless, the advent of TKIs has mostly replaced or used in combination with the use of standard chemotherapeutic agents (hydroxyurea, cyclophosphamide, vincristine, doxorubicin) depending upon the phase and outcome of the disease (Litzow et al., 2017; Pasic and Lipton, 2017). Despite the success with the frontline TKIs, and/or chemotherapy, where the complete molecular response can be achieved, there still remain a subset of CML patients especially the young who may require hematopoietic stem cell transplantation (HSCT) following induction and post-remission therapy (Litzow et al., 2017).

Interestingly, naïve or engineered mesenchymal stem cells (MSCs), isolated from different sources, such as the bone marrow, placenta, or umbilical cord, are reported to have anti-tumor effects (Chen et al., 2012; Tang et al., 2014). The umbilical cord-derived MSCs (UC-MSCs), when co-cultured with leukemic cells, induced cell cycle arrest (Chen et al., 2013); similarly, the use of the UC-MSCs *per se* or their extract inhibited cell viability and proliferation of Burkitt’s lymphoma cells (Lin et al., 2014). We have earlier identified that the MSCs derived from the Wharton’s jelly of the human umbilical cord (human Wharton’s jelly stem cells, hWJSCs) inhibit human breast and ovarian cancer cells (Gauthaman et al., 2012; Kalamegam et al., 2018). It is not known whether the anticancer effects of the hWJSCs are limited to a few cancers, and also there are no previous reports of its use against CML. Therefore, in the present study, we evaluated the effects of the hWJSCs and/or their extracts against the human chronic myeloid leukemic cell line (K562) *in vitro*, in terms of their viability, cell cycle, cell death, and related gene and protein expression.

## Materials and Methods

### Ethical Approval

The protocol for the derivation and use of the hWJSCs and the commercial human leukemic cell line was approved by the Bioethics Committee of the King Abdulaziz University *vide* approval number 33-15/KAU.

### Isolation and Culture of the hWJSCs

The human umbilical cords (hUCs) were collected following full-term delivery with informed patient consent from the Department of Obstetrics and Gynecology, King Abdulaziz University Hospital. The hWJSCs isolation and culture were done according to our earlier published studies (Fong et al., 2010; Gauthaman et al., 2012). Briefly, the UC was cut into ∼3 cm pieces and opened lengthwise. The tissue pieces were gently washed in sterile phosphate-buffered saline (PBS), and the opened side was placed facing down in a sterile plastic Petri dish (Greiner, Hamburg, Germany) containing enzymatic cocktail solution, comprising collagenase type I (2 mg/ml), collagenase type IV (2 mg/ml), and hyaluronidase (100 IU) (Sigma, MO, United States). The Petri dishes were then incubated at 37°C for 30 min in a 5% CO_2_ incubator (Life Technologies, Thermo Fisher Scientific, MA, United States) to facilitate the release of the hWJSCs. The cell suspension was then centrifuged (300 × *g*, 10 min), and the cell pellet was resuspended in the hWJSCs culture medium composed of 90% Dulbecco’s Modified Eagle’s Medium (DMEM) supplemented with 10% fetal bovine serum (FBS), 1% insulin–transferrin–selenium, 1% non-essential amino acids, 1% penicillin/streptomycin antibiotic mixture, and 16 ng/ml basic fibroblast growth factor (Life Technologies, Thermo Fisher Scientific, MA, United States). The hWJSCs were cultured in T25 cm^2^ tissue culture flasks (Greiner, Hamburg, Germany) and incubated at 37°C in a 5% CO_2_ incubator.

### Culture of the Human Chronic Myeloid Leukemia Cell Line (K562)

The commercial human leukemic cell line (K562) was purchased from the American Type Culture Collection (ATCC, MD, United States). The K562 cells were plated in a T25 cm^2^ tissue culture flask (Greiner; Hamburg, Germany) containing RPMI-1640 medium supplemented with 10% FBS and 1% penicillin/streptomycin mixture (Life Technologies, Thermo Fisher Scientific, MA, United States) and cultured in a humidified 5% CO_2_ incubator under standard culture conditions of 37°C and air atmosphere.

### Preparation of the hWJSC Extracts

The hWJSC extracts, namely, the hWJSC-conditioned medium (hWJSC-CM) and the hWJSC-lysate (hWJSC-L), were prepared according to an earlier published protocol (Gauthaman et al., 2012). Briefly, the hWJSC-CM was prepared from the early passages (P3–P4) of the hWJSCs (70–80% confluence) cultured in the hWJSCs medium for 48 h under standard culture conditions of 37°C and air atmosphere of 5% CO_2_. The medium was separated, filtered through a 0.22 mm Millex-GP syringe filter (Millipore, MA, United States), and stored at −20°C until use in experiments. The hWJSC-L was prepared by lysing the pelleted hWJSCs using 100 μl commercial mammalian cell lysis buffer (Cell Lytic, Sigma, MO, United States) and incubation on ice for 45 min. The lysate was centrifuged (15,000 rpm, 15 min), and aliquots of the supernatant were stored at −80°C until use in experiments. The total protein content of the hWJSC-L was measured using a Nanodrop^TM^ spectrophotometer (Nanodrop Technologies, DW, United States).

### MSC-Related CD Markers Expression

The surface CD markers expression in the hWJSCs was analyzed using flow cytometry as reported earlier (Kalamegam et al., 2018). Briefly, the hWJSCs (1 × 10^5^) were treated with different antibody cocktails (negative and positive) and incubated in the dark at 4°C for 30 min. The samples were washed twice with PBS containing 3% FBS and centrifuged (1,000 rpm, 5 min), and the pellet was resuspended in 500 μl of 3% FBS before analysis using FACS (FACS Aria III; BD Biosciences). The following antibodies (5 μl per CD marker; Miltenyi Biotec) were used: MSCs positive cocktail 1 (containing CD29-APC, CD90-FITC, and CD73-PERCP), MSCs positive cocktail 2 (containing CD44-APC and CD105-FITC), and MSCs negative cocktail 3 (containing CD34-APC and CD45-PE).

### Differentiation of the hWJSCs

The hWJSCs were differentiated into adipocytes, chondroblasts, and osteocytes using adipocytic (A10070-01), chondrocytic (A10071-01), or osteocytic (A10072-01) differentiation kits (StemPro^®^; Thermo Fisher Scientific) as reported earlier (Gari et al., 2016). Briefly, the hWJSCs (5 × 10^4^ cells/well) were seeded into three different 6-well plates and allowed to attach overnight, in the complete culture medium. The hWJSCs were then cultured in respective differentiation basal medium supplemented with appropriate supplement (StemPro^®^ kit content; Thermo Fisher Scientific) with fresh addition/change of medium every 72 h. The control cells were cultured using the differentiation basal medium alone. The hWJSCs that were differentiated into adipocytes, chondroblasts, and osteocytes were stained with oil red O, alcian blue, or alizarin red, respectively, according to the manufacturer’s instructions (Sigma, MO, United States) and imaged using light microscopy.

### Cell Morphology

Both the hWJSCs and the K562 cells were plated at a seeding density of 1 × 10^4^ cells, respectively, per well in a 24-well tissue culture plate for co-culture experiments. The K562 cells were plated at a seeding density of 2 × 10^4^ cells/well in a 24-well tissue culture plate for experiments with the hWJSC-CM (100%) and hWJSC-L (15 μg/ml). In all these experiments, the cells were cultured in a humidified 5% CO_2_ incubator at standard conditions of 37°C and air atmosphere for 24, 48, and 72 h. The morphological changes, if any, were imaged using a phase contrast microscope (Nikon ECLIPSE TS100, Japan).

### Cell Metabolic Activity (MTT Assay)

The K562 cells were plated as above, and the metabolic activity in the different experimental arms, namely, the hWJSCs co-culture (hWJSC-CC), hWJSC-CM (100%), and hWJSC-L (15 μg/ml), following culture for 24, 48, and 72 h was evaluated using MTT assay according to the manufacturer’s instructions. Briefly, at the end of the treatment period, 10 μl MTT reagent in 100 μl of fresh culture medium was added and incubated for 4 h at 37°C in a humidified incubator containing 5% CO_2_. The medium was removed, and 200 μl of dimethylsulfoxide (DMSO) (Sigma, MO, United States) was added to each well and incubated for 30 min in the dark to solubilize formazan crystals. The absorbance at 570 nm was measured using a spectrophotometer (SpectraMax i3 Multimode reader; Molecular Devices, United States).

### Cell Cycle (Propidium Iodide Assay)

The K562 cells were plated as above, and the cell cycle assay for the different experimental arms, namely, the hWJSC-CC, hWJSC-CM (100%), and hWJSC-L (15 μg/ml), was evaluated following culture for 48 h. The untreated K562 cells were used as a control. Briefly, the K562 cells were collected, washed in PBS thrice, and then fixed in ice-cold ethanol (70%) by dropwise addition to prevent cell clumps. The samples were stored at −20°C overnight. Before analysis, the fixed cells were centrifuged (1,000 rpm, 5 min) and washed twice with PBS. The cells were resuspended in 400 μl of staining solution containing propidium iodide (PI; 50 μg/ml) and RNAse (10 μg/ml). Following incubation in the dark for 15 min, the stained cells were analyzed using the FACS Aria III flow cytometer (BD Biosciences).

### Cell Death (Annexin V–APC and PI Staining Assay)

Both the hWJSCs and the K562 cells were plated at a seeding density of 1 × 10^4^ cells, respectively, per well in a 24-well tissue culture plate for co-culture experiments. The K562 cells were plated at a seeding density of 2 × 10^4^ cells/well in a 24-well tissue culture plate for experiments with the hWJSC-CM (100%) and hWJSC-L (15 μg/ml). The cell death assay, namely, Annexin V–allophycocyanin (APC) and PI, in the different experimental arms was evaluated according to the manufacturer’s instruction, following culture for 48 h. Briefly, the cells were washed with Annexin V binding buffer, stained with 5 μl Annexin V–APC at room temperature (RT) for 15 min, and then counterstained with PI (1 mg/ml). The cells were then centrifuged (1,000 rpm, 10 min), and the pellet was resuspended in 400 μl of Annexin V binding buffer and analyzed using flow cytometry (FACS Aria III; BD Biosciences).

### Gene Expression Analysis Using Real-Time Polymerase Chain Reaction

Gene expression following treatment of the K562 cells with either the hWJSC-CC, hWJSC-CM (100%), or hWJSC-L (15 μg/ml protein) and untreated controls at 48 h was evaluated using real-time polymerase chain reaction (qRT-PCR) as reported earlier (Kalamegam et al., 2018). Briefly, the total RNA was extracted using the RNeasy Mini Kit (Qiagen, Germany). First-strand cDNA was synthesized with random hexamers using a reverse transcriptase kit (Promega, WI, United States), and qRT-PCR was done using the SYBR Green master mix (Life Technologies, Thermo Fisher Scientific, MA, United States). The following genes related to inflammation (*IFN-γ*, *TNF-α*, *IL-1β*, *IL-4*, *IL-6*, *IL-8*, *IL-10*, and *IL-12A*) and cell death (*BAX*, *BCL2*, *BIRC5*, *CASP-3*) were analyzed using StepOnePlus^TM^ real-time PCR system (Thermo Fisher Scientific, MA, United States). The primer sequences were taken from earlier published studies and are given in Table 1.

**TABLE 1:** The genes and primer sequences used for real-time quantitative reverse transcription PCR.

Genes	Primer sequence
*GAPDH*	F: 5′-GCACCGTCAAGGCTGAGAAC-3′ R: 5′-GGATCTCGCTCCTGGAAGATG-3′
*IFN-γ*	F: 5′-CCCTCACACTCAGATCATCTTCT-3′ R: 5′-GCGTTGGACATTCAAGTCAG-3′
*TNF-α*	F: 5′-GGTGCTTGTTCCTCAGCCTC-3′ R: 5′-CAGGCAGAAGAGCGTGGTG-3′
*IL-1β*	F: 5′-CTGTCCTGCGTGTTGAAAGA-3′ R: 5′-TTGGGTAATTTTTGGGATCTACA-3′
*IL-6*	F: 5′-CCACTCACCTCTTCAGAA-3′ R: 5′-GCGCAAAATGAGATGAGT-3′
*IL-8*	F: 5′-AGACAGCAGAGCACACAAGC-3′ R: 5′-ATGGTTCCTTCCGGTGGT-3′
*IL-12A*	F: 5′-CACTCCCAAAACCTGCTGAG-3′ R: 5′-TCTCTTCAGAAGTGCAAGGGTA-3′
*IL-4*	F: 5′-TGGATCTGGGAGCATCAAGGT-3′ R: 5′-TGGAAGTGCGGATGTAGTCAG-3′
*IL-10*	F: 5′-GCTCTTACTGACTGGCATGAG-3′ R: 5′-CGCAGCTCTAGGAGCATGTG-3′
*BAX*	F: 5′-GGCTGGGATGCCTTTGTG-3′ R: 5′-CAGCCAGGAGAAATCAAACAGA-3′
*BCL-2*	F: 5′-TGGAGCTGCAGAGGATGATTG-3′ R: 5′-GCTGCCACTCGGAAAAAGAC-3′
*SURVIVIN*	F: 5′-ACCAGGTGAGAAGTGAGGGA-3′ R: 5′-AACAGTAGAGGAGCCAGGGA-3′
*CASPASE 3*	F: 5′-TGACTGGAAAGCCGAAACTC-3′ R: 5′-AGCCTCCACCGGTATCTTCT-3′

*F, forward primer; R, reverse primer.*

### Cytokine Analysis Using Multiplex Bead-Based Immunoassay

The cytokine analysis was performed using the Human Cytokine Magnetic bead-based assay (Thermo Fisher Scientific). The culture supernatant collected at 48 h following treatment of K562 with the hWJSC-CC, hWJSC-CM (100%), or hWJSC-L (15 μg/ml protein) was assayed as reported earlier (Kalamegam et al., 2018). Briefly, the various kit contents were prepared to working concentrations before use in the experiment. The 96-well plate was pre-wetted with 1× wash buffer, 25 μl/well of the antibody-coated polystyrene magnetic beads was added, and the beads were washed twice with 1× wash buffer. The standard (1:3 dilution) and undiluted samples were added to the beads and incubated at RT on an orbital shaker for 2 h to capture the analytes. Biotinylated detection antibodies (100 μl/well) were added and incubated for 1 h at RT and then with streptavidin-R-phycoerythrin antibodies (100 μl/well) for 30 min. The plate was washed twice with a wash buffer between incubations with different antibodies, using a hand-held magnetic bottom to safely retain the magnetic antibody beads. The beads were finally resuspended in wash buffer and analyzed using the Luminex MAGPIX^®^ instrument (Luminex Corporation, Austin, TX, United States). The data obtained were analyzed using the Luminex xPONENT^®^ multiplex assay analysis software (v.4.2.1324.0; Luminex Corporation).

### Statistical Analysis

Statistical analyses were performed using the Statistical Package for Social Sciences (SPSS) version 21. Students’ *t*-test or One-way ANOVA with Bonferroni *post-hoc* test was used for analysis between the control and treated groups. The values were expressed as mean ± standard error of the mean (SEM) from a minimum of three experimental replicates. Asterisk (^∗^) indicates the statistical significance of *P* < 0.05.

## Results

### Culture Morphology of the hWJSCs and K562 Cells

**FIGURE 1:**
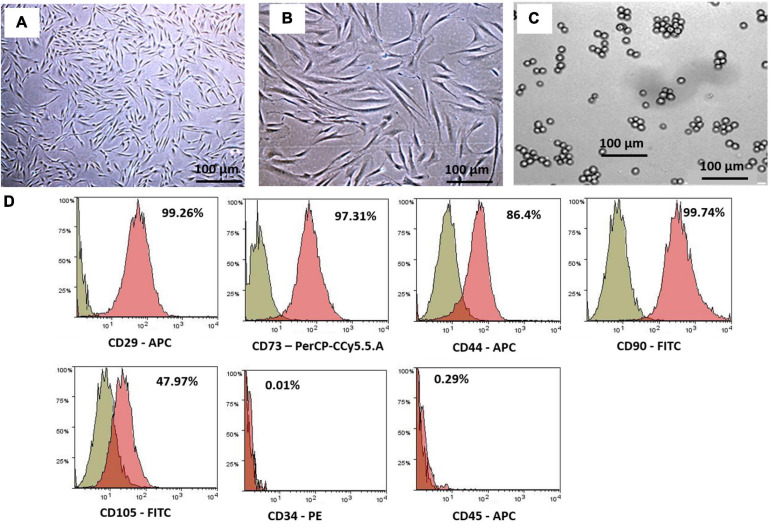
Phase contrast images showing primary cultures of the human Wharton’s jelly stem cells (hWJSCs) derived from the umbilical cord obtained following normal full-term delivery at **(A)** early passage (P0) and **(B)** later passage (P5). **(C)** Phase contrast image of the human chronic myeloid leukemia cell line (K562) in culutre. **(D)** Representative histogram of the CD surface markers on the hWJSCs using fluorescent activated cell sorting (FACS) analysis. The MSC positive surface CD markers, namely, CD29, CD73, CD44, CD90, and CD105, and the MSC negative surface CD markers, namely, CD34 and CD45, are shown. The independent CD marker antigens were tagged with different fluorochromes. All the MSC positive CD surface markers demonstrated more than 90% positivity. PE, phycoerythrin; APC, allophycocyanin; FITC, fluorescein isothiocyanate.

The hWJSCs were successfully isolated from all the umbilical cords and established in culture. Numerous colonies of the cells were observed in the primary cultures that formed confluent adherent cell monolayers from the first passage onwards. These cells resembled short fibroblasts in the initial cultures, and in the later passages, they appeared as long spindle-shaped fibroblasts (Figures 1A,B). The K562 cells grew as a suspension culture and showed rapid cell expansion. The K562 cells demonstrated their characteristic spherical shape resembling undifferentiated blast cells. These cells in suspension cultures appeared as either single or small clusters (5–7 cells) (Figure 1C).

### hWJSCs Express MSCs Positive CD Markers

The early passages of the hWJSCs analyzed showed more than 90% expression of MSC-related CD surface markers, namely, CD29 (98.2%), CD73 (94.0%), CD90 (99.4%), CD44 (98.7%), and CD105 (99.7%) (Figure 1D). In addition, the hWJSCs were negative for the hematopoietic markers, namely, CD34 (0.6%) and CD45 (0.2%) (Figure 1D). The expression of MSC-related CD markers at high percentages confirmed the stemness potential of the hWJSCs.

### hWJSCs Demonstrate Mesodermal Tissue Differentiation

**FIGURE 2:**
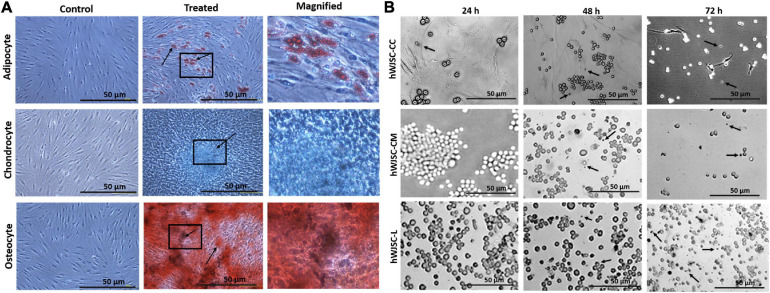
**(A)** *In vitro* differentiation images of the human Wharton’s jelly stem cells (hWJSCs) into adipocytes (top row), chondrocytes (middle row), and osteocytes (bottom row). The left column represents the respective controls, the middle column shows the differentiated images, and the right column is the magnified images of the boxed area. **(B)** Phase contrast images of the chronic myeloid leukemai cell line (K562) treated with the hWJSC co-culture (hWJSC-CC, top row), hWJSC-conditioned medium [hWJSC-CM (100%), middle row), and hWJSC lysate (hWJSC-L (15 μg/ml), bottom row] for 24, 48, and 72 h. A mild to moderate decrease in the K562 cells was eveident with an increase in cell death (black arrows) with time following treatment with the hWJSCs or their extracts.

The hWJSCs cultured in adipogenic, chondrogenic, and osteogenic differentiation media showed respective differentiation (Figure 2A). The hWJSCs cultured in adipocytic differentiation medium developed lipid vacuolations as early as 7 days of differentiation, and their numbers and size increased by day 21. These cells with vacuolations showed positive staining with oil red O (Figure 2A). The hWJSCs cultured in chondrocytic differentiation medium showed the transformation of spindle-shaped fibroblasts to more rounded chondroblast-like cells at day 21 that showed positive staining with alcian blue (Figure 2A) indicative of proteoglycans secretion in the extracellular matrix. In the osteogenic differentiation medium, the hWJSCs showed accumulation of granular deposits starting from day 14, and these cells showed positive staining with alizarin red (Figure 2A) indicative of calcium mineralization.

### hWJSCs Alter the Morphology of K562 Cells

The morphology of the K562 cells treated with the hWJSCs, hWJSC-CM, and hWJSC-L showed mild to moderate changes in cell morphology at different durations of culture, namely, 24, 48, and 72 h. Upon co-culture, the K562 cells settled onto the surface of the adherent hWJSCs and demonstrated variable changes in their morphology with some showing cell shrinkage, whereas others were enlarged compared with the control (Figure 2B, top row). The K562 cells treated with the hWJSC extracts in general showed enlarged cells that later were associated with membrane damage and cell death in both the hWJSC-CM (Figure 2B, middle row) and the hWJSC-L (Figure 2B, bottom row).

### hWJSCs Decrease the Metabolic Activity of K562 Cells

**FIGURE 3:**
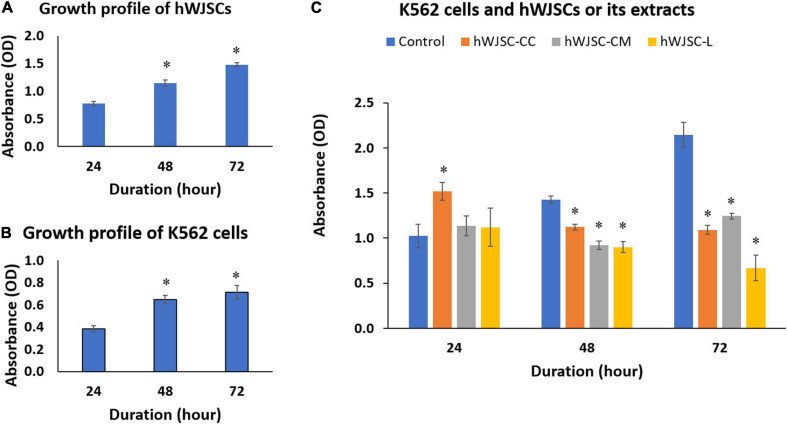
Cell metabolic activity (MTT) assay showing the cell growth profiles of **(A)** the human Wharton’s jelly stem cells (hWJSCs), **(B)** the chronic myeloid leukemai cell line (K562), and **(C)** the K562 cells treated with the hWJSC co-culture (hWJSC-CC), hWJSC-conditioned medium (hWJSC-CM, 100%), and hWJSC lysate (hWJSC-L, 15 μg/ml) for 24, 48, and 72 h. Mean increases in proliferation of the hWJSCs and K562 cells indicative of their biological cell growth characteristics were observed **(A,B)**. Mean decreases in proliferation of the K562 cells were observed following treatment with the hWJSCs and their extracts (hWJSC-CM, hWJSC-L) especially at 48 and 72 h, and these decreases were statistically significant compared with the control **(C)**. The values are expressed as mean ± SEM of three different experiments. ^∗^ indicates statistical significance (*P* < 0.05).

The biological baseline cultures of both the hWJSCs and the K562 cells showed an increase in the metabolic activity that indirectly reflects an increase in cell numbers with the duration of culture (Figures 3A,B). The increases in cell metabolic activity of the hWJSCs were 47.45 and 90.11% at 48 and 72 h compared with 24 h (Figure 3A). Similarly, the observed metabolic activities in the K562 cells were significantly increased by 24.54 (*P* = 0.018) and 57.48% (*P* = 0.0001), respectively, compared with 24 h (Figure 3B).

The K562 cells co-cultured with the hWJSCs (hWJSC-CC) demonstrated a significant increase in the cell metabolic activity at 24 h by 44.85% (*P* = 0.0001). In contrast, significant decreases in cell metabolic activity by 15.52 (*P* = 0.028) and 49.12% (*P* = 0.0001) were observed at 48 and 72 h, respectively, compared with their controls (Figure 3C). The K562 cells treated with the hWJSC-CM (100%) showed moderate to high decreases in cell metabolic activity by 30.42 and 41.98% at 48 and 72 h, respectively (Figure 3C). Similarly, the K562 cells treated with the hWJSC-L (15 μg/ml) also showed high decreases in cell metabolic activity by 32.23 and 68.80% at 48 and 72 h, respectively, compared with their controls (Figure 3C). All the above decreases in cell metabolic activity were statistically significant (*P* = 0.0001).

### hWJSCs Cause G2/M Arrest in K562 Cells

**FIGURE 4:**
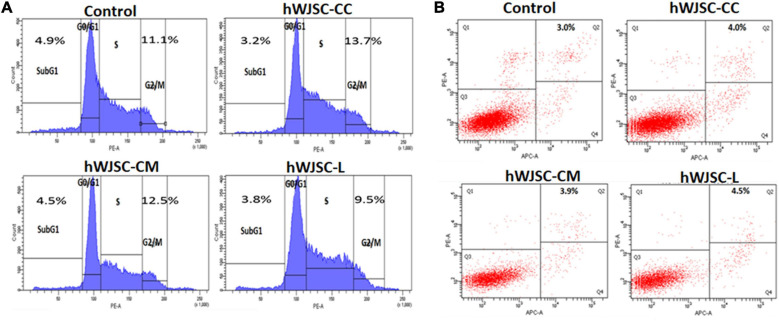
**(A)** Cell cycle (propidium iodide) assay. The K562 cells treated with the hWJSC co-culture (hWJSC-CC), hWJSC-conditioned medium (hWJSC-CM, 100%), and hWJSC lysate (hWJSC-L, 15 μg/ml) for 48 h showed an increase in the G2M phase of the cell cycle indicative of metaphase arrest. **(B)** Annexin V–APC and PI assay. The K562 cells treated as above with the hWJSCs and hWJSC extracts for 48 h showed an increase in the number of apoptosis cells compared with the control.

Cell cycle analysis of the K562 cells following treatment with the hWJSC-CC, hWJSC-CM (100%), and hWJSC-L (15 μg/ml) for 48 h demonstrated various changes in the sub-G1 and G2/M phases of the cell cycle (Figure 4A). The control (untreated) K562 cells showed normal cell cycle profiles. The K562 cells treated with the hWJSC-CC, hWJSC-CM, and hWJSC-L showed a decrease in the sub-G1 phase percentage by 3.2, 4.5, and 3.8%, respectively, compared with the control. There was an increase in the G2/M phase by 13.7 and 12.5% following treatment with the hWJSC-CC and hWJSC-CM (100%) compared with the control. In contrast, the K562 cells treated with the hWJSC-L (15 μg/ml) showed a decrease of 9.7% (Figure 4A).

### hWJSCs Induce Apoptosis in K562 Cells

Treatment of the K562 cells with the hWJSC-CC, hWJSC-CM (100%), and hWJSC-L (15 μg/ml) demonstrated cell death following incubation for 48 h (Figure 4B). The percentage of apoptotic cells was higher following treatment with the hWJSC-L (15 μg/ml) at 48 h compared with the hWJSC-CC and hWJSC-CM (100%). The K562 cells demonstrated apoptosis by 4.0, 3.9, and 4.5% with the hWJSC-CC, hWJSC-CM, and hWJSC-L, respectively, compared with the control (Figure 4B).

### hWJSCs Increase the Expression of Apoptotic Genes and Decrease the Inflammatory Genes

**FIGURE 5:**
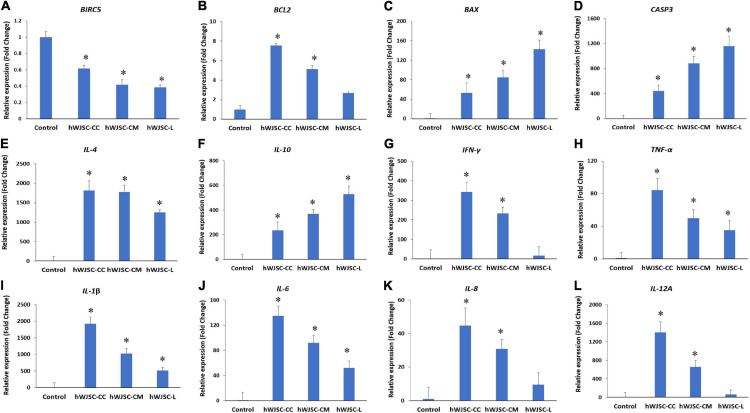
Gene expression analysis of the apoptosis- **(A–D)**, anti-inflammation- **(E,F)**, and pro-inflammation-related genes **(G–L)**, namely, *BIRC5*, *BCL2*, *BAX*, *CASP3*, *IL-4*, *IL-10 IFN-γ*, *TNF-α*, *IL-1β*, *IL-6*, *IL-8*, and *IL-12A*, in theK562 cells treated with the hWJSC co-culture (hWJSC-CC), hWJSC-conditioned medium (hWJSC-CM, 100%), and hWJSC lysate (hWJSC-L, 15 μg/ml) for 48 h, using quantitative real-time PCR. Data analysis and relative quantitation were performed using the comparative Ct method (ΔΔCt). ^∗^ indicates statistical significance (*P* < 0.05) compared with the control.

The K562 cells treated with the hWJSC-CC, hWJSC-CM (100%), and hWJSC-L (15 μg/ml) were evaluated for apoptosis- and inflammation-related genes. *BIRC5* (*Survivin*) showed a decrease in expression in the treated group compared with the control (*GAPDH*). The fold decreases in *Survivin* expression were 3.9, 5.8, and 6.2 for the hWJSC-CC, hWJSC-CM, and hWJSC-L, respectively (Figure 5A). The anti-apoptotic *BCL2* was increased compared with the control, but showed a declining trend with the hWJSC-CC (7.54-fold), hWJSC-CM (5.13-fold), and hWJSC-L (2.17-fold) in that order (Figure 5B). The pro-apoptotic *BAX* and *CASP3* were increased compared with the control. The fold increases in *BAX* were 52.78, 84.86, and 142.58, and the fold increases in *CASP3* were 442.55, 883.34, and 1,158.94 following treatment with the hWJSC-CC, hWJSC-CM, and hWJSC-L, respectively (Figures 5C,D).

The anti-inflammatory genes, namely, *IL-4* and *IL-10*, were increased compared with the control (*GAPDH*). The fold increases in *IL-4* were 1,814.46, 1,778.62, and 1,253.06, and the fold increases in *IL-10* were 235.07, 368.96, and 529.23 following treatment with the hWJSC-CC, hWJSC-CM, and hWJSC-L, respectively (Figures 5E,F). The following pro-inflammation-related genes, namely, *IFN-γ*, *TNF-α*, *IL-1β*, *IL-6*, *IL-8*, and *IL-12A*, were increased compared with the control, but showed a declining trend with the hWJSC-CC, hWJSC-CM, and hWJSC-L in that order (Figures 5G–L). The fold increases in gene expression were as follows: *IFN-γ* by 343.31, 233.48, and 17.00; *TNF-α* by 84.16, 49.98, and 35.21; *IL-1β by* 1,926.25, 1,029.87, and 516.32; *IL-6* by 134.94, 91.77, and 52.25; *IL-8* by 44.5, 30.87, and 9.51; and *IL-12A* by 1,402.18, 657.52, and 63.74 following treatment with the hWJSC-CC, hWJSC-CM, and hWJSC-L, respectively.

### Multiplex Cytokine Profile

**FIGURE 6:**
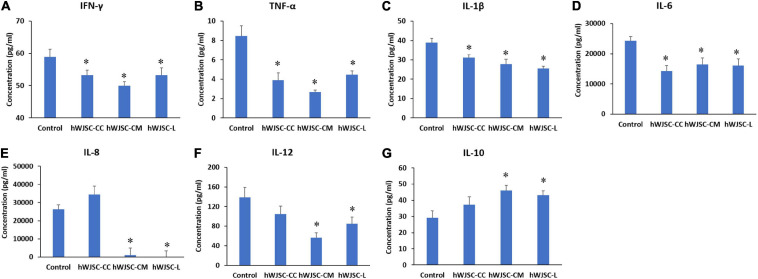
Expression levels of cytokines in the cell culture supernatant of the K562 cells treated with the hWJSC co-culture (hWJSC-CC), hWJSC-conditioned medium (hWJSC-CM, 100%), and hWJSC lysate (hWJSC-L, 15 μg/ml) for 48 h and analyzed using multiplex cytokine assay. The following pro-inflammation-related cytokines, namely, IFN-γ **(A)**, TNF-α **(B)**, IL-1β **(C)**, IL-6 **(D)**, IL-8 **(E)**, and IL-12 **(F)**, were decreased compared with the control. The anti-inflammatory cytokine IL-10 **(G)** was increased compared with the control. The values are expressed as mean ± SEM of three different experiments. ^∗^ indicates statistical significance (*P* < 0.05) compared with the control.

The secretion profile of some of the cytokines related to inflammation in addition to tumor invasion, progression, and migration, namely, IFN-γ, TNF-α, IL-1β, IL-6, IL-8, and IL-12, demonstrated a decrease in values following treatment with the hWJSC-CC, hWJSC-CM (100%), and hWJSC-L (15 μg/ml) (Figures 6A–F). The mean decreases in values were as follows: IFN-γ by 9.70, 15.17, and 9.70%; TNF-α by 54.15, 68.36, and 47.3%; IL-1β by 20.15, 28.48, and 34.50%; IL-6 by 41.27, 32.17, and 34.22%; and IL-12 by 24.47, 59.47, and 38.51% following treatment with the hWJSC-CC, hWJSC-CM, and hWJSC-L, respectively. IL-8 showed an increase by 30.57% (hWJSC-CC) and decreases by 96.01 (hWJSC-CM) and 99.47% (hWJSC-L) (Figure 6E). IL-10 showed a mean percentage increase by 27.09, 57.30, and 47.35% following treatment with the hWJSC-CC, hWJSC-CM, and hWJSC-L, respectively (Figure 6G).

## Discussion

The erythroleukemia cell line K562, carrying the genetic abnormality of *BCR-ABL1*, derived from a pleural effusion of a female patient in blast crisis (Lozzio and Lozzio, 1975) is one of the most commonly used models for research on malignant hematopoiesis and molecular pathogenesis of leukemia. The presence of the oncogenic tyrosine kinase *BCR-ABL1* leads to the activation and self-proliferation of the stem cell population and causes the dysregulation of the apoptotic pathways. Most commonly, the TKIs that target the *BCR-ABL* oncogenic pathways remain the frontline of CML therapy, which despite being effective initially, led to the later emergence of drug resistance. The presence of T3151 mutation is reported to be associated with the development of drug resistance and reduction in the efficacy of TKIs especially imatinib, and this subset of T3151 mutation was identified to be present in 7% of CML (Chahardouli et al., 2013).

Different forms of therapies are used against leukemia depending upon the type and stage of the disease; however, transplantation of stem cells continues to remain an important option. Allogenic HSCT has been used in the management of leukemic disorders for long, with variable success. Recently, the use of umbilical cord blood transplantation (UCBT) for leukemic patients who may require an allograft, as an alternative in the absence of an HLA-matched donor, is reported. However, the limitations observed with the widespread use of UCBT are in part due to their delay in the replenishment of the hematopoietic microenvironment and the immune competency to overcome the original disorder (Algeri et al., 2020). The MSCs, apart from their use as cell-based therapies in regenerative medicine, are reported to have both pro- or anti-cancer properties, either in their naïve state or after being engineered to carry apoptotic inducers or small molecules (Hmadcha et al., 2020; Wu et al., 2020).

In the present study, we have utilized the K562 cell line to study the anti-cancer properties of the hWJSCs *in vitro* using many independent assays. The derived hWJSCs demonstrated the expression of MSC-related CD surface markers (Figure 1D) and readily differentiated into mesodermal tissue lineage, such as adipocytes, osteocytes, and chondroblasts (Figure 2A). The cell morphology, where early passages resembled short fibroblasts and epithelioid cells and later passages showed long spindle-shaped fibroblast-like cells (Figures 1A,B), as well as their differentiation potential, was in line with earlier studies (Subramanian et al., 2015; Balgi-Agarwal et al., 2018). The MSCs including the hWJSCs have been used to replenish the depleted marrow or differentiated along the blood cell lineages for therapeutic applications against various blood disorders (Lin et al., 2014). We observed that the hWJSCs and their extracts (hWJSC-CM, hWJSC-L) caused cell shrinkage and membrane damage in the K562 cells leading to their death (Figure 2B) as well as their inhibition (Figure 3C). Lin et al. (2014) reported similar morphological changes and inhibition profile in the human Burkitt’s lymphoma cell line treated with the hUC-MSCs and their extracts (Lin et al., 2014). Besides, the hWJSCs and/or their extracts are reported to inhibit breast, bone, and ovarian cell lines *in vitro* mainly due to their paracrine effects (Chao et al., 2012; Kalamegam et al., 2018).

Cell cycle dysregulation is a hallmark of cancerous cells, and therefore arresting the progression of cell cycle or induction of apoptosis are considered as some important strategies for cancer therapeutics (Hanahan and Weinberg, 2011). We observed that the hWJSCs and hWJSC extracts caused cell cycle arrest of the K562 cells in the G2/M phase (Figure 4A). Interestingly, the G2/M phase has a checkpoint that signifies a potential target for cancer therapy. The cells that have damaged DNA in the late “S” or “G2” phases are prevented from entering the mitotic phase, thus halting the cell cycle progression (Wang et al., 2009). A recent study has reported an increase in the number of the K562 cells following co-culture with rat bone marrow-derived MSCs in the G0/G1 phase of the cell cycle, coupled with decrease in the “S” and “G2/M” phases indicating the inhibition of cell cycle progression (Fathi et al., 2019).

Tumor cells overcome the host surveillance and continue their survival by inhibiting apoptosis, and therefore considerable interest has been directed to understand the signaling mechanisms that impair or promote apoptosis in leukemia and thus guide novel therapeutics targeting apoptotic genes or pathways (Yang et al., 2016). We observed that the hWJSCs and/or their extracts inhibited the K562 cells by induction of apoptosis in addition to causing cell cycle arrest. The percentage of inhibition was more evident with the hWJSCs and hWJSC-L than with the hWJSC-CM (Figure 4B). Interestingly, Lin et al. (2014) demonstrated the ability of the hWJSCs and their extracts to induce apoptosis in lymphoma cells. Entosis as a mechanism for internalization of the hWJSCs during co-culture leading to the death of MDA-MB-231 breast cancer cells by lysosomal involvement has been reported (Chao et al., 2012). It is possible that the hWJSCs in the present study induced apoptosis by a similar mechanism with co-culture or by activating the cell death signaling mechanisms by the hWJSC secretions; however, further studies are necessary to conclusively prove this notion.

In line with the FACS analysis, we also observed an increase in the pro-apoptotic *BAX* and *CASPASE 3* following treatment compared with the control (Figures 5C,D). Caspases are known to be involved in apoptosis, and that activation of caspase-3/7 results in DNA fragmentation (Cohen, 1997). A recent study also reported that exosomes released by the UC-MSCs sensitize the K562 cells to imatinib induction of apoptosis *via* activation of the caspases (Liu et al., 2018). The same authors, however, noted that the exosomes from the hUC-MSCs by itself did not have any inhibition of the K562 cells proliferation nor induced apoptosis by itself. The hWJSCs are specifically isolated from within the Wharton’s jelly matrix of the umbilical cord, and it is understood that there are MSCs from different compartments of the umbilical cord with varying stemness potentials (Subramanian et al., 2015). Therefore, it yet remains to be understood whether the cancer inhibitory effects of the MSCs depend on their inherent properties and/or their origin/source from where these stem cells are derived. The observed increase in *BAX* gene expression in the treated K562 cells indicates the altered balance of pro-survival to pro-apoptotic signaling mechanisms leading to the execution of the K562 cells. The *SURVIVIN* gene expression was also decreased in the K562 cells following exposure to the hWJSCs and their extracts (Figure 5A), thus impeding the survival of the K562 cells. *SURVIVIN* encoded by the *BIRCH 5* gene located at chromosome 17 is known to inhibit apoptosis by (i) acting upon caspases, (ii) stabilizing the apoptosis inhibitor proteins, and (iii) regulating the apoptotic protease-activating factor 1 release from the mitochondria (Dohi et al., 2004; Sah et al., 2006). The anti-apoptotic *BCL2* was higher than the control, which is quite expected in most cancer cell lines, but had a decreasing trend in the treated groups. Further studies will help us to better understand whether the BH3 only proteins of the *BCL2* family are expressed following treatment with the hWJSCs and/or their extracts, which may then interact downstream with *BAX* leading to caspase activation and apoptosis.

To understand whether the hWJSCs might bring about the K562 cells inhibition *via* secretion of cytokines, we analyzed its secretory profile using multiplex bead-based cytokine analysis. We observed that the anti-inflammatory cytokine IL-10 was increased following treatment with the hWJSCs and their extracts (Figure 6G). The Th1 and Th2 cytokines produced by the CD4+ and CD8+ T cells might bring about the destruction of the CML cells. However, there was no reported increase in the Th1 or Th2 cell response in CML patients compared with healthy volunteers (Kiani et al., 2003). The present increase in IL-10 following the hWJSCs and their extracts may help either in priming the cytotoxic T helper cells and/or in overcoming the T cell anergy when administered alone or in combination with other immunological agents.

Chronic inflammation supports tumor initiation and progression, in part due to various soluble and cellular inflammatory mediators that allow tumor cells to escape immune surveillance. In the present study, we also observed that the pro-inflammatory cytokines that promote cancer progression, namely, IFN-γ, TNF-α, IL-1β, IL-6, IL-8, and IL-12, were decreased (Figures 6A–F). An earlier study that evaluated the association between proinflammatory cytokines and cancer incidence revealed that IL-1β, IL-6, and TNF-α are associated with an increased risk of cancer (Trompet et al., 2009). However, it is important to understand that IL-6 and IL-8 help restore the hematopoietic activity of the bone marrow that becomes altered in CML and provides support to maintain the normal turnover of stem cells/progenitor cells (Shi et al., 2018). Dysregulation of the cytokine microenvironment in the bone marrow niche supports the progression of leukemia and also impairs normal hematopoietic stem cell turnover. In an elegant high-content screening study using a library of 313 cytokines, the positive regulators of primitive CML cells were screened, and it was identified that IFN-γ, IL-1β, and IL-6 had growth-promoting effects along with IL-3, IL-1α, and granulocyte-macrophage colony-stimulating factor (GM-CSF) as reported earlier (von Palffy et al., 2020). Interestingly, the authors also identified five novel cytokines that were hitherto not reported but were associated with the growth-promoting properties of CML. They are myostatin propeptide, sCD-14, IL-21, Il-13v, and CCL-28. Of these, myostatin propeptide was identified to be the most potent in promoting the growth of CML primitive cells (von Palffy et al., 2020).

## Conclusion

The present study analyzed the inhibitory effects of the hWJSCs and their extracts against the CML cell line (K562) *in vitro*. We observed that the hWJSCs and their extracts decreased the K562 cell numbers, by inducing cell cycle arrest and causing apoptosis. We also identified that the hWJSCs/hWJSC extracts upregulated the anti-inflammatory cytokines and downregulated the pro-inflammatory genes as well as the cytokines that probably led to the inhibition of the K562 cells. The emergence of other mutations and persistence of resistance of CML cells against existing management strategies have led researchers to look for novel therapeutic agents that might help remission. Given the beneficial role of HSCT when other therapeutic modalities fail or become less effective, it will be strategic to use the hWJSCs that carry both embryonic and MSC properties to help not only in the regeneration of the defective marrow but also in causing inhibition and cell death of the abnormal blast cells. However, further in-depth proteomics and metabolomics studies are necessary to identify the definitive molecule(s) and understand their cancer cell inhibitory mechanisms.

## Data Availability Statement

All datasets generated for this study are included in the article/supplementary material, further inquiries can be directed to the corresponding author/s.

## Ethics Statement

The ethical approval for derivation and the use of the human Wharton’s jelly stem cells (hWJSCs) and the commercial human leukemic cell line was approved by the Bioethics Committee of the King Abdulaziz University *vide* approval number 33-15/KAU.

## Author Contributions

GK, MH, and PP were involved in the conceptualization, intellectual contribution, statistical evaluation, and manuscript writing. KS is a clinician and was involved in providing the clinical materials/information and intellectual support. MH, RK, GA, FA, and GK were involved in the experimental work and data analysis. TQ and PP were involved in the co-ordination of the work, review, and editing of the manuscript. All authors contributed to the article and approved the submitted version.

## Conflict of Interest

The authors declare that the research was conducted in the absence of any commercial or financial relationships that could be construed as a potential conflict of interest.

## Abbreviations

ABL: Abelson
ANOVA: Analysis of variance
APC: Allophycocyanin
BAX: BCL-2-associated X
BCL2: B-cell lymphoma 2
BCR: Breakpoint cluster region
BM-MSCs: Bone marrow mesenchymal stem cells
CD: Cluster of differentiation
CML: Choric myelogenous leukemia
COX-2: Cyclooxygenase-2
FACS: Fluorescent-activated cell sorting
HSCT: Hematopoietic stem cell transplantation
hWJSC-CM: Human Wharton’s jelly stem cells—conditioned medium
hWJSC-L: Human Wharton’s jelly stem cells—lysate
hWJSCs: Human Wharton’s jelly stem cells
IFN-γ: Interferon-gamma
IL: Interleukin
MTT: 3-(4,5-dimethylthiazol-2-yl)-2,5-diphenyl tetrazolium bromide
PE: Phycoerythrin
PECy7: Phycoerythrin and a cyanine dye
PERCP: Peridinin–chlorophyll–protein
SEM: Standard error of the mean
TKIs: Tyrosine kinase inhibitors
TNF-α: Tumor necrosis factor-alpha
UC-MSCs: Umbilical cord mesenchymal stem cells.

## References

[B1] Algeri M. Gaspari S. Locatelli F. (2020). Cord blood transplantation for acute leukemia. *Expert Opin. Biol. Ther.* 20 1223–1236. 10.1080/14712598.2020.1782380 32529854

[B2] American Cancer Society (2015). *Cancer Facts & Figures 2015.* Atlanta, GA: American Cancer Society.

[B3] Balgi-Agarwal S. Winter C. Corral A. Mustafa S. B. Hornsby P. Moreira A. (2018). Comparison of preterm and term Wharton’s jelly-derived mesenchymal stem cell properties in different oxygen tensions. *Cells Tissues Organs* 205 137–150. 10.1159/000489256 29949803 PMC6117836

[B4] Bawazir A. Al-Zamel N. Amen A. Akiel M. A. Alhawiti N. M. Alshehri A. (2019). The burden of leukemia in the Kingdom of Saudi Arabia: 15 years period (1999–2013). *BMC Cancer* 19:703. 10.1186/s12885-019-5897-5 31315607 PMC6637507

[B5] Bonifacio M. Stagno F. Scaffidi L. Krampera M. Di Raimondo F. (2019). Management of chronic myeloid leukemia in advanced phase. *Front. Oncol.* 9:1132. 10.3389/fonc.2019.01132 31709190 PMC6823861

[B6] Bonnet D. Dick J. E. (1997). Human acute myeloid leukemia is organized as a hierarchy that originates from a primitive hematopoietic cell. *Nat. Med.* 3 730–737. 10.1038/nm0797-730 9212098

[B7] Chahardouli B. Zaker F. Mousavi S. A. Kazemi A. Ostadali M. Nadali F. (2013). Evaluation of T315I mutation frequency in chronic myeloid leukemia patients after imatinib resistance. *Hematology* 18 158–162. 10.1179/1607845412Y.0000000050 23540562

[B8] Chao K. C. Yang H. T. Chen M. W. (2012). Human umbilical cord mesenchymal stem cells suppress breast cancer tumourigenesis through direct cell–cell contact and internalization. *J. Cell. Mol. Med.* 16 1803–1815. 10.1111/j.1582-4934.2011.01459.x 21973190 PMC3822693

[B9] Chen F. Zhou K. Zhang L. Ma F. Chen D. Cui J. (2013). Mesenchymal stem cells induce granulocytic differentiation of acute promyelocytic leukemic cells via IL-6 and MEK/ERK pathways. *Stem Cells dev.* 22 1955–1967. 10.1089/scd.2012.0621 23391335 PMC3685326

[B10] Chen Q. Cheng P. Song N. Yin T. He H. Yang L. (2012). Antitumor activity of placenta-derived mesenchymal stem cells producing pigment epithelium-derived factor in a mouse melanoma model. *Oncol. Lett.* 4 413–418. 10.3892/ol.2012.772 23741242 PMC3673651

[B11] Cohen G. M. (1997). Caspases: the executioners of apoptosis. *Biochem. J.* 326 1–16. 10.1042/bj3260001 9337844 PMC1218630

[B12] Collins S. J. Groudine M. T. (1983). Rearrangement and amplification of c-abl sequences in the human chronic myelogenous leukemia cell line K-562. *Proc. Natl. Acad. Sci. U.S.A.* 80 4813–4817. 10.1073/pnas.80.15.4813 6308652 PMC384135

[B13] Dohi T. Beltrami E. Wall N. R. Plescia J. Altieri D. C. (2004). Mitochondrial survivin inhibits apoptosis and promotes tumorigenesis. *J. Clin. Invest.* 114 1117–1127. 10.1172/JCI22222 15489959 PMC522254

[B14] Fathi E. Farahzadi R. Valipour B. Sanaat Z. (2019). Cytokines secreted from bone marrow derived mesenchymal stem cells promote apoptosis and change cell cycle distribution of K562 cell line as clinical agent in cell transplantation. *PLoS One* 14:e0215678. 10.1371/journal.pone.0215678 31009502 PMC6476492

[B15] Fong C. Y. Subramanian A. Biswas A. Gauthaman K. Srikanth P. Hande M. P. (2010). Derivation efficiency, cell proliferation, freeze-thaw survival, stem-cell properties and differentiation of human Wharton’s jelly stem cells. *Reprod. Biomed. Online* 21 391–401. 10.1016/j.rbmo.2010.04.010 20638335

[B16] Gari M. Alsehli H. Gari A. Abbas M. Alkaff M. Abuzinadah M. (2016). Derivation and differentiation of bone marrow mesenchymal stem cells from osteoarthritis patients. *Tissue Eng. Regen. Med.* 13 732–739. 10.1007/s13770-016-0013-2 30603454 PMC6170872

[B17] Gauthaman K. Yee F. C. Cheyyatraivendran S. Biswas A. Choolani M. Bongso A. (2012). Human umbilical cord Wharton’s jelly stem cell (hWJSC) extracts inhibit cancer cell growth in vitro. *J. Cell. Biochem.* 113 2027–2039. 10.1002/jcb.24073 22275115

[B18] Goldman J. M. Melo J. V. (2003). Chronic myeloid leukemia—advances in biology and new approaches to treatment. *N. Engl. J. Med.* 349 1451–1464. 10.1056/NEJMra020777 14534339

[B19] Hanahan D. Weinberg R. A. (2011). Hallmarks of cancer: the next generation. *Cell* 144 646–674. 10.1016/j.cell.2011.02.013 21376230

[B20] Hmadcha A. Martin-Montalvo A. Gauthier B. R. Soria B. Capilla-Gonzalez V. (2020). Therapeutic potential of mesenchymal stem cells for cancer therapy. *Front. Bioeng. Biotechnol.* 8:43. 10.3389/fbioe.2020.00043 32117924 PMC7013101

[B21] Kabarowski J. H. S. Witte O. N. (2000). Consequences of BCR-ABL expression within the hematopoietic stem cell in chronic myeloid leukemia. *Stem Cells* 18 399–408. 10.1002/stem.180399 11072027

[B22] Kalamegam G. Sait K. H. W. Ahmed F. Kadam R. Pushparaj P. N. Anfinan N. (2018). Human Wharton’s Jelly stem cell (hWJSC) extracts inhibit ovarian cancer cell lines OVCAR3 and SKOV3 in vitro by inducing cell cycle arrest and apoptosis. *Front. Oncol.* 8:592. 10.3389/fonc.2018.00592 30581772 PMC6293270

[B23] Kiani A. Habermann I. Schake K. Neubauer A. Rogge L. Ehninger G. (2003). Normal intrinsic Th1/Th2 balance in patients with chronic phase chronic myeloid leukemia not treated with interferon-alpha or imatinib. *Haematologica* 88 754–761. 12857553

[B24] Lin H. D. Fong C. Y. Biswas A. Choolani M. Bongso A. (2014). Human Wharton’s jelly stem cells, its conditioned medium and cell-free lysate inhibit the growth of human lymphoma cells. *Stem Cell Rev. Rep.* 10 573–586. 10.1007/s12015-014-9514-3 24789672

[B25] Litzow M. R. Ak Fielding S. M. Luger E. Paietta Y. Ofran J. M. Rowe A. H. (2017). The evolving role of chemotherapy and hematopoietic cell transplants in Ph-positive acute lymphoblastic leukemia in adults. *Bone Marrow Transplant.* 52 1592–1598. 10.1038/bmt.2017.110 28581459

[B26] Liu Y. Song B. Wei Y. Chen F. Chi Y. Fan H. (2018). Exosomes from mesenchymal stromal cells enhance imatinib-induced apoptosis in human leukemia cells via activation of caspase signaling pathway. *Cytotherapy* 20 181–188. 10.1016/j.jcyt.2017.11.006 29269240

[B27] Lozzio C. B. Lozzio B. B. (1975). Human chronic myelogenous leukemia cell-line with positive Philadelphia chromosome. *Blood* 45 321–334. 10.1182/blood.V45.3.321.321 163658

[B28] Nowell P. C. Hungerford D. A. (1960). Chromosome studies on normal and leukemic human leukocytes. *J. Natl. Cancer Inst.* 25 85–109. 14427847

[B29] Pasic I. Lipton J. H. (2017). Current approach to the treatment of chronic myeloid leukaemia. *Leuk. Res.* 55 65–78. 10.1016/j.leukres.2017.01.005 28135648

[B30] Sah N. K. Khan Z. Khan G. J. Bisen P. S. (2006). Structural, functional and therapeutic biology of survivin. *Cancer Lett.* 244 164–171. 10.1016/j.canlet.2006.03.007 16621243

[B31] Shi H. Wang Y. Li R. Xing W. Yang F. C. Bai J. (2018). Alteration in the cytokine secretion of bone marrow stromal cells from patients with chronic myelomonocytic leukemia contribute to impaired hematopoietic supportive activity. *Stem Cells Int.* 2018 5921392. 10.1155/2018/5921392 30123289 PMC6079359

[B32] Subramanian A. Fong C. Y. Biswas A. Bongso A. (2015). Comparative characterization of cells from the various compartments of the human umbilical cord shows that the Wharton’s jelly compartment provides the best source of clinically utilizable mesenchymal stem cells. *PLoS One* 10:e0127992. 10.1371/journal.pone.0127992 26061052 PMC4464659

[B33] Tang X. J. Lu J. T. Tu H. J. Huang K. M. Fu R. Cao G. (2014). TRAIL-engineered bone marrow-derived mesenchymal stem cells: TRAIL expression and cytotoxic effects on C6 glioma cells. *Anticancer Res.* 34 729–734. 24511006

[B34] Trompet S. de Craen A. J. M. Mooijaart S Stott D. J. Ford I Sattar N. (2009). High innate production capacity of proinflammatory cytokines increases risk for death from cancer: results of the PROSPER study. *Clin. Cancer Res.* 15 7744–7748. 10.1158/1078-0432.CCR-09-2152 19996221

[B35] von Palffy S. Landberg N. Sandén C. Zacharaki D. Shah M. Nakamichi N. (2020). A high-content cytokine screen identifies myostatin propeptide as a positive regulator of primitive chronic myeloid leukemia cells. *haematologica* 105 2095–2104. 10.3324/haematol.2019.220434 31582541 PMC7395258

[B36] Wang H. Naghavi M. Allen C. Barber R. M. Bhutta Z. A. Carter A. (2016). Global, regional, and national life expectancy, all-cause mortality, and cause-specific mortality for 249 causes of death, 1980–2015: a systematic analysis for the global burden of disease study 2015. *Lancet* 388 1459–1544. 10.1016/S0140-6736(16)31012-1 27733281 PMC5388903

[B37] Wang Y. Ji P. Liu J. Broaddus R. R. Xue F. Zhang W. (2009). Centrosome-associated regulators of the G_2_/M checkpoint as targets for cancer therapy. *Mol. Cancer* 8:8. 10.1186/1476-4598-8-8 19216791 PMC2657106

[B38] Wu Z. Liu W. Wang Z. Zeng B. Peng G. Niu H. (2020). Mesenchymal stem cells derived from iPSCs expressing interleukin-24 inhibit the growth of melanoma in the tumor-bearing mouse model. *Cancer Cell Int.* 20 1–10. 10.1186/s12935-020-1112-7 32015693 PMC6990536

[B39] Yang K. Wang X. Zhang H. Wang Z. Nan G. Li Y. (2016). The evolving roles of canonical WNT signaling in stem cells and tumorigenesis: implications in targeted cancer therapies. *Lab. Invest.* 96, 116–136. 10.1038/labinvest.2015.144 26618721 PMC4731283

